# Lipopolysaccharide-induced inflammation or unilateral ureteral obstruction yielded multiple types of glycosylated Lipocalin 2

**DOI:** 10.1186/s12950-016-0116-5

**Published:** 2016-03-05

**Authors:** Yoko Fujiwara, Hiroyoshi Tsuchiya, Nobuya Sakai, Katsushi Shibata, Akio Fujimura, Taka-aki Koshimizu

**Affiliations:** Division of Molecular Pharmacology, Department of Pharmacology, Jichi Medical University, Tochigi, 329-0498 Japan; Department of Functional Genomics, Graduate School of Pharmaceutical Sciences, Himeji Dokkyo University, Hyogo, 670-8524 Japan; Division of Clinical Pharmacology, Department of Pharmacology, Jichi Medical University, Tochigi, 329-0498 Japan

**Keywords:** Lipocalin 2, N-glycan, Urinary tract obstruction, Lipopolysaccharide, Glycoprotein, Urine

## Abstract

**Background:**

The amount of urinary glycoprotein lipocalin 2 (LCN2) has been known to increase after kidney injury because of failed reabsorption by the proximal tubules or direct secretion from injured tissues. However, the relationship between urinary tract obstruction and the isoform diversity of LCN2 has not been examined.

**Methods:**

The urinary levels of LCN2 isoforms were examined in male mice after an intraperitoneal injection of lipopolysaccharide (LPS) or in a mouse model of unilateral ureter obstruction (UUO). The LCN2 levels in sera, bladder urine, renal pelvic urine, and tissue samples were also analyzed. Endo- and exoglycosidases were used to investigate the different N-glycan patterns of LCN2.

**Results:**

Two isoforms of urinary LCN2 with different molecular weights were identified in an immunoblotting analysis, and the levels of both isoforms were increased 6 h after LPS administration. The primary LCN2 isoform was the lower molecular weight 22-kDa isoform, which was detected in the serum, urine, liver and kidney. In contrast, the 24-kDa LCN2 isoform was detected only in urine. In the UUO experiments, the levels of the 24-kDa LCN2 were increased in the bladder urine but not in the urine accumulated in the renal pelvis due to UUO. The 22-kDa LCN2 was identified in the renal pelvic urine from UUO mice. The peptide-N glycosidase F digestion of the two urinary LCN2 isoforms generated a single protein. Moreover, the two urinary LCN2 proteins were sensitive to neuraminidase and resistant to endoglycosidase H (Endo H). The LCN2 in the serum, lung and kidney was resistant to Endo H, as observed in urine, whereas the LCN2 in the liver and the ureter were degraded by this enzyme.

**Conclusions:**

These results suggest that the difference in the molecular weights of the LCN2 proteins was due to their N-glycan structure. The high molecular weight LCN2 in urine could be detected after the inflammatory response to LPS and UUO. Furthermore, the sensitivity to Endo H identified the presence of two types of carbohydrate moieties, depending on the tissue in which the LCN2 was produced. These findings are useful for widening the clinical applicability of urinary LCN2 analyses.

## Background

Lipocalins constitute a family of small secreted proteins with a common structure, primarily consisting of an eight-stranded beta-sheet, that form hydrogen-bonded beta-barrels [1]. The members of this family serve different functions by binding to small hydrophobic ligands and play an important role in regulating the immune system activity [1]. Lipocalin 2 (LCN2), which is also called neutrophil gelatinase-associated lipocalin (NGAL) or 24p3, has been identified in a variety of samples, including neutrophils, uterine luminal fluid, and SV40-infected primary kidney cells [2–4]. Many lipocalins act as a carrier for specific proteins and fatty acids, and the LCN2 proteins can form complexes with catecholate-type siderophores, such as enterochelins, which are small iron-containing molecules [5]. These LCN2-iron complexes bind to cell surface receptors to transport iron into the cell [6]. The sequestration of iron by LCN2 could play a protective role in the innate immune response against bacterial infections and may be involved in cellular processes, such as the induction of apoptosis [5–7].

During the acute phase response, LCN2 is significantly upregulated [8]. It has been investigated as a non-invasive biomarker for a variety of diseases, including inflammation and acute kidney injury [9–12]. Under physiological conditions, expression of the *Lcn2* gene can be detected in the trachea, bone marrow, colon, lungs, prostate, and uterus in humans [4, 13]. However, *Lcn2* gene expression and protein synthesis are enhanced in various cells under stressful conditions, such as cancer, acute kidney or liver injury, infection, and ulcerative colitis [9, 14–17]. The bacterial endotoxin LPS induces the transcription of *Lcn2* via toll-like receptor 4 [18]. Additionally, LPS often induces a variety of cytokines [19–21]. Proinflammatory cytokines, including interleukin-1β (IL-1β) and IL-17, in combination with tumor necrosis factor α (TNFα), can facilitate the promoter activity of the *Lcn2* gene [17, 22–24]. These cytokines activate the transcription factor nuclear factor-kappa B (NF-κB) by stabilizing the cofactor IκB-ζ transcript, which is essential for the induction of *Lcn2* gene transcription [22, 24].

The LCN2 proteins produced at sites of inflammation are rapidly secreted into the systemic circulation, pass through the glomeruli, and are completely reabsorbed by renal proximal tubular cells via the scavenger-receptor megalin and cubilin-dependent endocytosis [25–28]. Increased urinary LCN2 levels suggest proximal tubule dysfunction, which contributes to failed urinary LCN2 reabsorption, or the *de novo* synthesis of LCN2 in the injured kidney [29, 30]. For example, Kuwabara et al. reported that an increase in urinary LCN2 in streptozotocin-induced diabetic mice was caused by a dysfunction of tubular reabsorption, and elevated serum LCN2 levels were considered to be a source of urinary LCN2 [27]. Predicting the origin of urinary LCN2 may help to identify proximal tubule dysfunction distinct from direct injury of the urinary tract, but such knowledge is limited at present.

We previously established an animal model of drug-induced kidney injury, which was accompanied by urinary LCN2 elevation [31]. In the present study, we characterized two subpopulations of LCN2 with distinct molecular weights, 22- and 24-kDa LCN2, in the urine from model mice with LPS-induced inflammation or ureteral obstruction. Because LCN2 is a member of the glycoprotein family [4, 32], the 24-kDa urinary LCN2 was expected to be more glycosylated than 22-kDa LCN2. In the current study, our deglycosylation analysis revealed that the LCN2 in the serum, urine, and peripheral tissues exhibited different N-glycan patterns in LPS-treated mice. Moreover, the 24-kDa form of LCN2 could be detected in urine from mice with experimental unilateral ureteral obstruction. Our results suggest that the characterization of the glycoforms of LCN2 may help to narrow down their origin, and it may be possible to link certain glycoforms of LCN2 to specific organ dysfunction.

## Methods

### Materials

Dulbecco’s modified Eagle’s medium (DMEM), LPS (*E. coli* O128:B12), and dexamethasone (Dex) were obtained from Sigma-Aldrich (St. Louis, MO, USA). Fetal bovine serum, antibiotics, and cloning vectors were purchased from Life Technologies (Carlsbad, CA, USA). HEK293 cells were obtained from the American Type Culture Collection (Manassas, VA, USA). The anti-mouse LCN2 antibody was purchased from Abnova (Taipei, Taiwan). Goat anti-rabbit IgG was obtained from GE Healthcare Life Sciences (Buckinghamshire, UK). Complete Mini, EDTA-free protease inhibitor cocktail was from Roche (Mannheim, Germany). The DNA ligation kit and EX Taq were purchased from Takara Bio (Shiga, Japan). PNGase F, Endo H, and Neuraminidase (NA) were obtained from New England BioLabs (Herts, UK). Coomassie brilliant blue (CBB) solution and the Rapid Stain CBB kit were procured from Nacalai Tesque (Kyoto, Japan). All other chemicals were purchased from Wako Pure Chemical Industries (Osaka, Japan).

### Animals

Male and female BALB/c CrSlc and male C57BL/6J JmsSlc mice were obtained from SLC Japan (Shizuoka, Japan). All animals used in this study were eight weeks old. The mice were housed in micro-isolator cages in a pathogen-free barrier facility (12 h light: 12 h darkness cycle) with access to regular chow and water *ad libitum*. All animal experiments were approved by the Animal Care and Use Committee of Jichi Medical University.

### Drug administration

Male and female BALB/c CrSlc and male C57BL/6J JmsSlc mice were intraperitoneally (i.p.) injected with 1 mg/kg of LPS. Saline was administered as a control. Dex (5 mg/kg) or vehicle (saline) was administered i.p. 1 h prior to LPS. After 6 h, the mice were euthanized by cervical dislocation, and trunk blood samples were collected and allowed to clot for 30 min at room temperature, followed by centrifugation at 3000 × *g* for 10 min to obtain serum. The tissues were sampled and homogenized in lysis buffer (50 mM Tris–HCl (pH 7.4), 150 mM NaCl, 1 % Nonidet P40, 1 mM EDTA (pH 8.0), 1 mM EGTA, and 1 mM phenylmethylsulfonyl fluoride) containing a protease inhibitor cocktail and were centrifuged at 12,000 × *g* for 5 min to collect the supernatant. The protein concentrations were measured using the CBB method. Spot urine samples were collected and immediately placed on ice, and urine, serum, and tissue lysates were stored at −80 °C until further use. For the measurement of the serum C-related protein (CRP) level, serum samples were diluted 2000–20,000-fold and CRP and IL-1β levels were measured using ELISA kits (R & D Systems, Minneapolis, MN, USA). The absorbance at 450 nm and 540 nm was measured using a SpectraMax Pro M3 instrument (Molecular Device, CA, USA).

### UUO experiments

For the UUO experiments, the mice were anesthetized with 50 mg/kg of pentobarbital sodium i.p. After laparotomy, the left ureter was ligated with a silk suture (5–0) 0.7 cm below the renal pedicle. Three days later, the mice were euthanized by cervical dislocation, then their urine, which accumulated in the obstructed side of the kidney and bladder, was sampled by syringe aspiration with 30 G 1/2 and 26 G 1/2 needles. Control mice underwent a sham operation (the ureter was manipulated but not ligated). Urine samples were kept at −80 °C until use.

### Preparation of recombinant mouse LCN2

The coding sequence for mouse LCN2 cDNA was amplified from brain cDNA by PCR using Ex Taq reagents. The primer sequences used to amplify mouse *Lcn2* were 5′-AAACCATGGCCCTGAGTGTCAT-3′ and 5′-GCCTGAACCATTGGGTCTCTGC-3′. The PCR products were subcloned into the pCR-4 vector according to the manufacturer’s protocol and sequenced. The mouse LCN2 cDNA was then ligated into the pcDNA3.1 expression vector. Human embryonic kidney (HEK293) cells were cultured in a six-well dish with DMEM containing 10 % heat-inactivated fetal bovine serum, 100 U/ml penicillin, and 100 μg/ml streptomycin at 37 °C in 5 % CO_2_. Plasmid DNA was transfected into HEK293 cells with the FuGENE HD transfection reagent (Promega, Madison, WI) following the manufacturer’s protocol. The culture media were exchanged to serum-free media 24 h after transfection and was collected 48 h after transfection. Five hundred microliters of culture medium were further centrifuged at 14,000 × *g* for 10 min at 4 °C for concentration and was replaced with phosphate-buffered saline (PBS) using Amicon Ultra Centrifugal Filter Devices (10 K molecular weight cutoff, Millipore, Billerica, MA, USA). The buffer exchange was repeated three times. The concentrated media, the final volume of which was approximately 60 μL, were prepared for immunoblot and glycosylation analyses.

### Gel electrophoresis and immunoblotting

Protein samples were heated with sodium dodecyl sulfate (SDS) buffer (10 % glycerol, 2.3 % SDS, 5 % 2-mercaptoethanol, 62.5 mM Tris–HCl (pH 6.8), and 0.1 % bromophenol blue) at 100 °C for 5 min. Spot urine (2 μg of protein), tissues (5 μg), sera (5 μg), and glycosidase-digested samples (5 μg of proteins and 2 μL of concentrated-conditioned media) were electrophoresed on 12.5 % SDS-polyacrylamide gels and transferred to polyvinylidene difluoride membranes. Urine samples from UUO mice (2 μL of urine from the renal pelvis and 5 μg of bladder urine (approximately 5–10 μL)) were also subjected to electrophoresis. After blocking (Block Ace; DS Pharma Biomedical, Osaka, Japan) for 1 h at room temperature, the membranes were incubated with a rabbit polyclonal antibody specific for mouse LCN2, which was diluted 1:8000 with Can Get Signal Immunoreaction Enhancer Solution (TOYOBO, Osaka, Japan) overnight at 4 °C. After incubation for 1 h at room temperature with horseradish peroxidase-labeled goat anti-rabbit IgG, which was diluted 1:10,000 as a secondary antibody, the membranes were treated with ECL Western blotting detection reagents (Perkin Elmer Life Sciences, Boston, MA) and visualized with a LAS-3000 fluorescence imaging system (Fuji Film, Tokyo, Japan).

### Quantitative analysis for gene expression

Tissue samples were isolated from LPS-treated male BALB/c mice, and total RNA was extracted using TRIzol reagent (Life Technologies, Carlsbad, CA). To synthesize first-strand cDNA, 0.5 μg of total RNA was reverse transcribed with the PrimeScript RT reagent kit (Takara Bio, Shiga, Japan) per the manufacturer’s protocol. Gene-specific primers were used for quantitative real-time PCR as follows: mouse *Lcn2*–5′-CTGAATGGGTGGTGAGTGTG-3′ for the left primer and 5′-GGGAGTGCTGGCCAAATAAG-3′ for the right primer; mouse β-actin–5′- TTGCTGACAGGATGCAGAAG-3′ for the left primer and 5′-ACATCTGCTGGAAGGTGGAC-3′ for the right primer. The reactions were performed on a Dice Thermal Cycler using SYBR Premix Ex Taq II (Takara Bio, Shiga, Japan). The expression levels of β-actin were used as an internal control.

### Glycosylation analysis

Sera, urine, concentrated conditioned media, tissue samples, and proteins used as positive controls (fetuin and RNase B) were treated with PNGase F, Endo H, or NA according to the manufacturer’s instructions. PNGase F hydrolyzes between the innermost N-acetylglucosamine (GlcNAc) and Asn residues of all types of N-glycan. Endo H cleaves between the two GlcNAc residues of the chitobiose core of hybrid- or high mannose-type N-glycan, and NA digests the terminal N-acetyl-neuraminic acid residues from carbohydrates [33, 34]. Ten micrograms of protein and 9 μL of conditioned media were glycosidase digested overnight at 37 °C. The samples were then boiled in SDS-sample buffer for 5 min and analyzed by immunoblotting. SDS-polyacrylamide gels loaded with fetuin and RNase B were stained with CBB after electrophoresis.

### Quantitative image analysis and statistical analysis

The images were analyzed using the ImageJ gel analysis software program (http://imagej.nih.gov/ij/). The data are expressed as the mean ± S.E.M. Significant differences were evaluated using an unpaired *t*-test or two-way ANOVA. For multiple comparisons, *P* values were adjusted by Holm’s method. *P* < 0.05 was considered significant.

## Results

### Detection of the two forms of LCN2 in urine

**Fig. 1:**
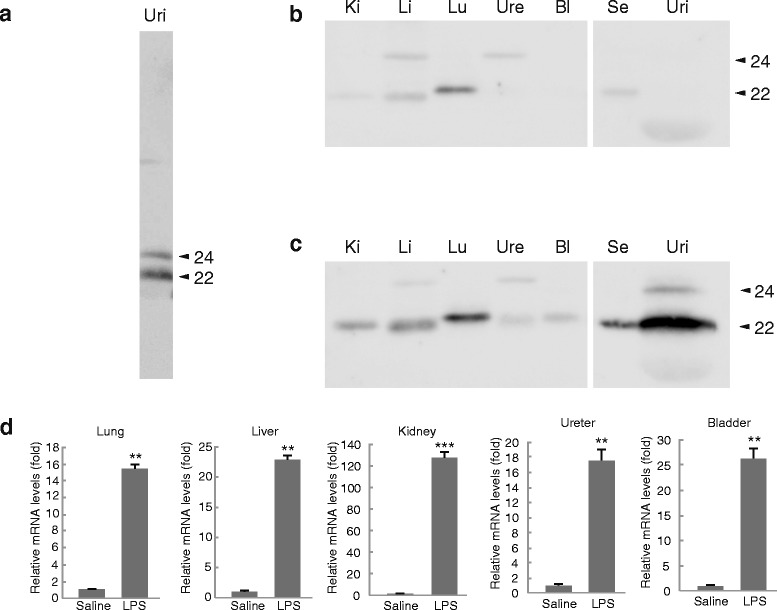
Detection of LCN2 in the urine from LPS-treated mice. Representative results of a Western blot analysis of the urinary LCN2 from LPS-treated mice (**a**). Male BALB/c mice were treated with (**b**) saline or (**c**) LPS (1 mg/kg i.p.) for 6 h. Tissue, serum, and urinary protein samples of 5 μg were subjected to Western blot analyses. Lu: lung, Li: liver, Ki: kidney, Ure: ureter, Bl: bladder, Se: serum, and Uri: urine. The numbers on the right sides of the figures indicate the molecular size of LCN2 (kDa). *n* = 6–8 for saline, and *n* = 8–10 for LPS treatment. (**d**) The LCN2 mRNA expression levels in peripheral tissues. The expression was normalized relative to that of β-actin, which served as an internal control. *n* = 5–7 for saline, and *n* = 4–5 for LPS treatment. Error bars represent the S.E.M. ** *P* < 0.01, and *** *P* < 0.001

Previous studies reported that LPS challenge drastically increased the LCN2 expression at the protein and mRNA levels in the rat lung, liver, and kidney, as well as in the mouse lung and liver [35, 36]. Our present work also showed that LPS administration increased the LCN2 mRNA and protein expression in the ureter and bladder, as well as in the lung, liver and kidney of the BALB/c mice (Fig. 1). Figure 1a shows a representative result demonstrating two different isoforms of urinary LCN2. Urinary LCN2 was hardly detectable under basal conditions, but was detectable as a signal of apparently stronger intensity compared with that of serum LCN2 after the administration of LPS (Fig. 1b and c). While the LCN2 protein in the serum from LPS-treated mice was a single band with a molecular weight of 22 kDa, the urinary LCN2 proteins in these mice showed two forms with different molecular weights, i.e., 22 and 24 kDa (Fig. 1c).

In peripheral tissues, the majority of the upregulated LCN2 was approximately 22 kDa in size. Faint LCN2-immunopositive signals, which were slightly larger than the 24-kDa urinary LCN2, were also detected in the liver and ureter in the saline-injected mice. However, LPS did not increase the densities of this band in either type of sample. The single and double bands of LCN2 in the serum and urine, respectively, were also observed in LPS-treated female BALB/c and male C57B6 mice, indicating that the sample-specific molecular patterns of LCN2 proteins did not exhibit sex- or strain-specific differences (data not shown). These results suggest that the urinary LCN2 in LPS-treated mice consists of two distinct molecular weight forms. Notably, the 24-kDa LCN2 was upregulated in the urine, but not in the serum, by LPS treatment.

### The temporal expression of urinary LCN2 proteins

**Fig. 2:**
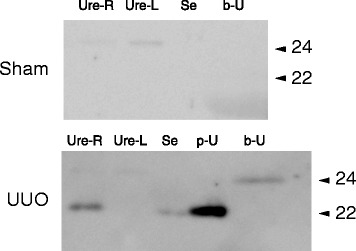
Detection of two LCN2 glycosylated forms in urine after three days of UUO. Representative images showing the LCN2 protein expression in the right (*obstructed*) and left (*intact*) ureters, sera, and urine samples collected from the obstructed side of the renal pelvis or bladder in UUO mice. Each experiment was repeated six times for the sham operation and 10 times for the UUO mice. Ure-R, right ureter; Ure-L, left ureter; Se, serum; p-U, urine in renal pelvis; and b-U, bladder urine. The numbers on the right side of the figures indicate the molecular size of LCN2 (kDa). Renal pelvic urine (p-U) could be obtained only after ureteral obstruction was established. Therefore, the results for pelvic urine are only shown for the UUO group

Because the 24-kDa LCN2 protein was specifically observed in the urine of LPS-treated mice, we hypothesized that it may serve as a biomarker to identify urinary tract disorders. To prove this hypothesis, we established UUO mice as a model of a urinary tract disorder. To search for the origin of the 24-kDa LCN2 in urine, we performed Western blot analyses of serum, urine and tissue samples from these model mice. In the UUO experiments, unilateral ureteral ligation successfully upregulated the 22-kDa LCN2 expression on the obstructed side of the ureter without affecting the opposite side (Fig. 2). An immunopositive signal for 24-kDa LCN2 was barely detectable in both ureters of sham-operated and UUO mice. The serum level of the 22-kDa LCN2 was increased in UUO mice. Despite the faint LCN2 band at 24 kDa, a robust signal for LCN2 was detected at 22 kDa in the urine that had pooled in the renal pelvis (Fig. 2). Interestingly, the 24-kDa LCN2 was observed only in the bladder urine of UUO mice, with significant differences in the signal-to-noise ratio (1.06 ± 0.012 for the sham operation, *n* = 6, and 1.35 ± 0.037 for UUO, *n* = 10, *P* = 0.047 based on an unpaired *t*-test). These results suggest that a urinary tract obstruction upregulates the urinary 24-kDa LCN2 level, which is predominantly found in bladder urine.

### Two glycosylation patterns of urinary LCN2

**Fig. 3:**
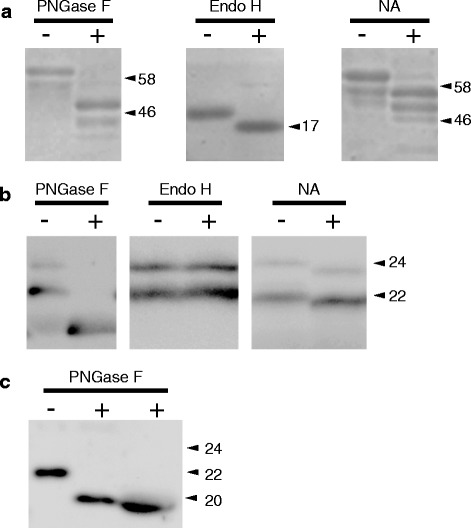
Endo- and exoglycosidase digestions of urinary LCN2 from LPS-treated mice. (**a**) Proteins were subjected to SDS-PAGE and CBB staining. Fetuin (48 kDa) served as a positive control substrate for PNGase F and NA, whereas RNase B (17 kDa) was the control substrate for Endo H. Each experiment was repeated three times. The numbers on the right sides of the figures indicate the molecular marker size (kDa). (**b**) The N-glycosylation sites of mouse urinary LCN2 were digested by PNGase F, Endo H, or NA. Each experiment was repeated six times. The numbers on the right sides of the figures indicate the molecular size of LCN2 (kDa). (**c**) Comparison of the PNGase F digestions between recombinant mouse LCN2 (*left and center*) and native mouse urinary LCN2 (*right*). Each experiment was repeated three times. Protein samples (5 μg) and samples of conditioned media (2 μL) were resolved by gel electrophoresis. The numbers on the right sides of the figures indicate the molecular size of LCN2 (kDa)

Because a previous study reported that mouse LCN2 contains two N-glycosylation sites in its amino acid sequence [4], we postulated that the two LCN2 forms with different molecular weights observed in our experiment were the result of post-transcriptional N-glycosylation. The positive control assays in which fetuin and RNase B were added as substrates for the endo- or exoglycosidase showed enzymatic activity (Fig. 3a). Using urinary LCN2 from LPS-treated mice as a substrate, PNGase F digestion produced a single molecular weight protein (Fig. 3b). The product size of the PNGase F-digested urinary LCN2 was nearly equal to the molecular weight of unglycosylated-recombinant mouse LCN2, i.e., 20 kDa (Fig. 3c). The two forms of urinary LCN2 proteins remained unaffected by Endo H, suggesting that the urinary LCN2 proteins had neither high-mannose nor hybrid type N-glycan (Fig. 3b). In addition, neuraminidase (NA), which digests terminal N-acetyl-neuraminic acid residues from carbohydrates, shifted the immunopositive bands of each urinary LCN2 to a lower molecular weight. These results indicate that the 22- and 24-kDa proteins observed in the Western blotting analysis contained the same LCN2 protein and complex-type N-glycan with terminal N-acetyl-neuraminic acid, but the structures of their carbohydrate moieties differed.

### The glycosylated forms of the LCN2 proteins in the peripheral tissues

**Fig. 4:**
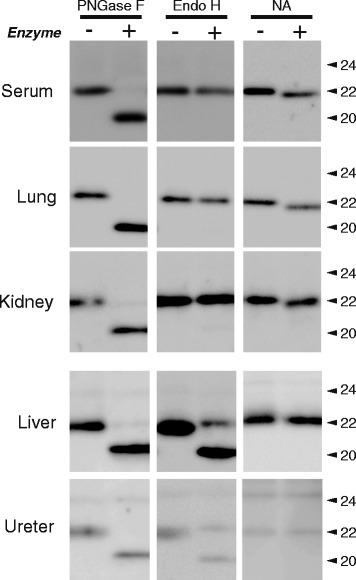
The glycosidase sensitivities of LCN2 in the serum and peripheral tissues. LCN2 in the serum, lungs, kidneys, liver, and ureters from LPS treated-mice was treated with endo- and exoglycosidases (PNGase F, Endo H, and NA). Each experiment was repeated three to five times, and 5 μg of protein was resolved by gel electrophoresis. The numbers on the right sides of the figures indicate the molecular size of LCN2 (kDa)

When mice were treated with LPS, increases in the levels of LCN2 (at approximately 22 kDa) were detected in the peripheral tissues (Fig. 1). Interestingly, the molecular weight of the LCN2 expressed in peripheral tissues subtly differed among tissues. For example, the mass of LCN2 was slightly higher in the lung than in the liver. To investigate the features of the carbohydrate moieties of LCN2 in the serum and tissues, we examined the glycopeptidase sensitivity of the LCN2 proteins in representative tissues obtained from LPS-treated mice. Similar to observations in urinary samples, the molecular weights of the LCN2 proteins from all examined tissues shifted to lower values (approximately 20 kDa) after PNGase F treatment (Fig. 4). However, in contrast to urinary LCN2, Endo H was partially effective in releasing N-glycan from the 22-kDa LCN2 in the liver and the ureter, whereas this protein minimally cleaved the carbohydrates on LCN2 from the sera, lungs, and kidneys of LPS-treated mice (Fig. 4). NA treatment clearly affected the LCN2 digestion in the serum and Endo H-resistant tissues, such as the lung and the kidney, whereas it minimally influenced the size of LCN2 in Endo H-sensitive tissues, such as the liver and the ureter of LPS-treated mice. These results indicate that the sensitivities of LCN2 proteins to glycosidases differed between the urine and certain tissues, and the LCN2 proteins secreted in the serum and urine, as well as in the lungs and kidneys, of LPS-treated mice mainly contain complex N-glycans.

### The correlation between LCN2 and pro-inflammatory markers

**Fig. 5:**
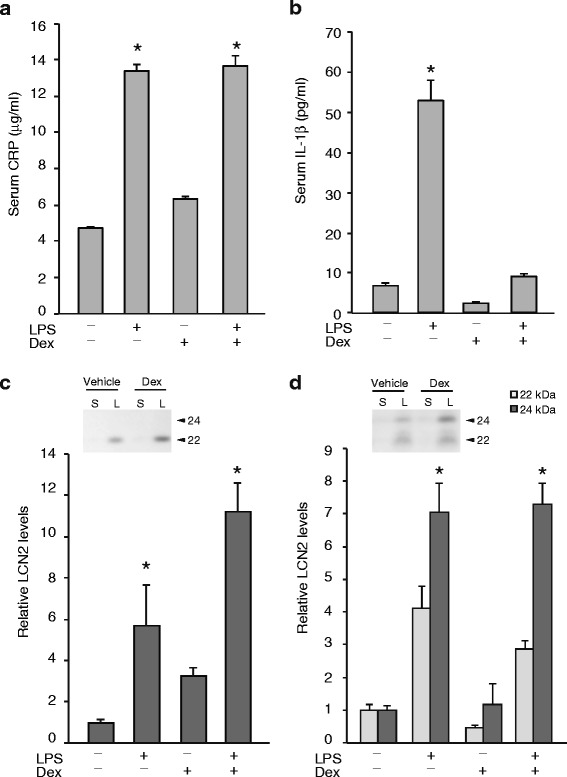
Dexamethasone pretreatment did not prevent the LPS-induced increase in LCN2. The serum and urinary LCN2 levels were examined after pretreatment with a vehicle (saline) or dexamethasone (Dex, 5 mg/kg) 1 h prior to the administration of saline or LPS (1 mg/kg i.p.). The LPS treatment significantly increased serum CRP (**a**) and IL-1β (**b**) levels. Dexamethasone pretreatment suppressed an increase of serum IL-1β, but not of CRP. The serum (**c**) and urinary (**d**) LCN2 protein levels were significantly increased by LPS treatment (*p* < 0.05). However, Dex treatment did not change the serum or urinary LCN2 levels. The values shown were the signal intensities relative to those of the samples from mice administered the vehicle and saline. S, saline treatment; and L, LPS treatment. (*n* = 5) *, *P* <0.05 vs. saline

We then measured the levels of serum pro-inflammatory markers, such as IL-1β, in our LPS-treated model to evaluate the correlation of inflammatory responses with the production of urinary LCN2 isoforms. In addition, the effects of steroid pretreatment (Dex; 5 mg/kg) on the LCN2 production following LPS treatment was investigated. LPS administration (1 mg/kg i.p.) actively caused systemic inflammation, which increased the serum CRP levels in both the saline- and Dex-pretreated mice (Fig. 5a, *P* < 0.001 for LPS treatment, *P* = 0.28 for Dex treatment, and *P* = 0.42 for LPS × Dex treatment, by a two-way ANOVA).

LPS increased the serum IL-1β level in the saline-pretreated mice (Fig. 5b). In contrast, Dex pretreatment suppressed the production of serum IL-1β upon LPS exposure (Fig. 5b). Despite the effective suppression of the serum IL-1β levels by Dex pretreatment, the serum and urinary LCN2 levels were significantly increased after LPS treatments (Fig. 5c and d). Thus, the upregulation of LCN2 by LPS treatment correlated with pro-inflammatory cytokine and CRP elevation, but the LCN2 production in our model could not be suppressed by pretreatment with 5 mg/kg Dex.

## Discussion

The present study revealed that there are two distinct types of urinary LCN2 isoforms that are induced after LPS stimulation or UUO manipulation. The 24-kDa urinary LCN2 was more glycosylated than the 22-kDa form. Specifically, it contained an N-glycosyl side chain that differed from the glycosylated 22-kDa LCN2 found in the serum and peripheral tissues. Previous studies also indicated that the urinary LCN2 can be separated into several isoforms in animal models of drug-induced kidney injury [31] and in mice with diabetic nephropathy or ischemia-reperfusion injuries [9, 27]. Multiple LCN2-immunopositive proteins have also been found in conditioned media from human endometrial cancer cell lines and in plasma samples from patients with chronic myelogenous leukemia [37, 38]. Although the differences in the two forms of LCN2 were not the result of glycosylation in the case of chronic myelogenous leukemia [38], our results suggest that multiple glycoforms of urinary LCN2 can be generated by changing the attached N-glycans. The previous studies and our present works indicate that the inflammatory actions associated with various diseases are significant events leading to the production of multiple LCN2 forms.

Although LCN2 can bind siderophores to act as an iron carrier, the differences in the molecular weight of LCN2 observed in urinary samples are not attributable to LCN2-siderophore complexes because the molecular weight of a siderophore in mammals is less than 1 kDa and the siderophore binds to LCN2 in a 1:1 ratio [7, 39, 40].

Our results revealed that different tissues show different distributions of LCN2 that are dependent on the in vivo glycosylation patterns. Namely, 22-kDa LCN2 was widely distributed throughout the body, including the serum and urine, whereas the heavier 24-kDa form was found in the bladder urine from the UUO model mice. In general, circulatory LCN2 is filtered freely through the glomerular membrane and is reabsorbed by proximal tubular cells via the cell surface receptor, megalin [26]. In a mouse model of acute endotoxemia, LPS downregulated the megalin receptor expression [41], suggesting that there was failed reabsorption by the proximal tubular cells. In our present study, the estimated molecular weight and glycosidase sensitivity were similar between the urinary 22-kDa LCN2 and the serum LCN2. Thus, the upregulation of urinary LCN2, especially 22-kDa LCN2, could be partly attributed to the leakage of abundant circulatory LCN2 due to the dysfunction of the reabsorption mechanism resulting from megalin downregulation. We also considered that the urinary 22-kDa LCN2 might be directly secreted from the kidney, ureter, and bladder due to its overexpression. However, we only detected 24-kDa LCN2 in the urine, not in the serum. This isoform may originate from the serum by glomerular filtration, but its origin remains unclear. The detailed mechanism(s) underlying the production of the heavily glycosylated urinary 24-kDa LCN2 require further study.

The distribution patterns of urinary 22- and 24-kDa LCN2 varied between the LPS-treated and ureteral obstruction model mice, suggesting that the emergence of the two different glycosylated urinary LCN2 isoforms could differ according to the pathological conditions. We hypothesize that the urinary 22-kDa LCN2 accumulated around the obstructed renal pelvis in our UUO mice was mainly derived from the damaged kidney and injured ureter. Interestingly, ureteral obstruction predominantly induced the 24-kDa LCN2 isoform in bladder urine, which was derived from the unobstructed side of kidney and ureter. Ureteral obstruction can damage renal cortical tubular cells and result in interstitial fibrosis due to macrophage infiltration, generating pro-inflammatory cytokines, such as TNF-α, on the obstructed side of the kidney [42–44]. Other cytokines, such as IL-1β or IL-17, which are involved in stimulating *Lcn2* gene expression, are also induced by UUO [45, 46]. Thus, the urinary 24-kDa LCN2 in the UUO model primarily arose from the damaged ureter on the obstructed side. In humans, the level of LCN2 is significantly higher in the bladder urine of patients with ureteropelvic junction obstruction (UJPO) than in healthy subjects [47]. The glycoforms of LCN2 are thus expected to be useful to identify urinary tract injuries, such as UJPO.

The LCN2 isoforms detected in the mouse serum, urine and some peripheral tissues were divided into two types depending on their sensitivities to glycosidases. We observed that there were differences between Endo H and NA sensitivity in the carbohydrate moiety of LCN2 in individual peripheral tissues. Namely, Endo H-resistant and NA-sensitive LCN2 was detected in the lungs and kidneys of the LPS-treated mice. Conversely, in the livers and ureters of these mice, Endo H treatment clearly cleaved the N-glycan of LCN2, whereas the mobility of LCN2 after NA treatment was not changed. Therefore, the N-linked carbohydrate moieties of LCN2 in the liver and ureter are predicted to have a high mannose or hybrid-type content and a lower sialylated N-glycan content than the Endo H-resistant LCN2, such as the isoforms found in the lungs and kidneys. In our experiments, the secreted LCN2 in the mouse serum and urine contained the Endo-H-resistant and NA-sensitive complex N-glycans, whereas Endo-H-sensitive LCN2 could not be detected in the serum or urine of LPS-treated mice.

The structure of N-glycan is involved in the apical sorting of secretory glycoproteins in epithelial cells, and removal of the terminal sialic acids from N-glycan leads to a shorter plasma half-life for the glycoprotein [48, 49]. Therefore, carbohydrate moieties may regulate the extracellular delivery or serum stability of LCN2 proteins. Further investigations will help to clarify the structure of N-glycan and the secretory properties of LCN2.

## Conclusions

Our results indicate that LCN2 can exist in multiple glycosylated forms, depending on the type of injury present and the type of sample examined. For example, the more glycosylated urinary LCN2 isoform could be useful for predicting urinary tract obstruction. Further studies of the glycoforms of LCN2 will provide valuable insight into the correlation between the glycosylation of LCN2 and various pathological conditions.

## Abbreviations

CBB: Coomassie brilliant blue
CRP: C-related protein
Dex: dexamethasone
DMEM: Dulbecco’s modified Eagle’s medium
Endo H: endoglycosidase H
GlcNAc: N-acetylglucosamine
HEK293: human embryonic kidney cells
i.p: intraperitoneally
IL: interleukin
LCN2: lipocalin 2
LPS: lipopolysaccharide
NA: neuraminidase
NF-κB: nuclear factor-kappa B
NGAL: neutrophil gelatinase-associated lipocalin
PBS: phosphate-buffered saline
PNGase F: peptide-N glycosidase F
SDS: sodium dodecyl sulfate
TNF-α: tumor necrosis factor α
UJPO: ureteropelvic junction obstruction
UUO: unilateral ureteral obstruction
